# Carp (Cyprinidae) Fisheries in Swedish Lakes: A Combined Environmental Assessment Approach to Evaluate Data-limited Freshwater Fish Resources as Food

**DOI:** 10.1007/s00267-019-01241-z

**Published:** 2019-12-19

**Authors:** Sara Hornborg, Anton Främberg

**Affiliations:** 1 RISE- Research Institutes of Sweden, PO Box 5401, 402 29 Gothenburg, Sweden; 2 Department of Biological and Environmental Sciences, University of Gothenburg, Gothenburg, Sweden

**Keywords:** Environmental assessment, Life cycle assessment, Productivity susceptibility analysis, Freshwater fisheries, Cyprinidae, Food security

## Abstract

The role of aquatic resources to food security is both promising and constrained since the global seafood consumption is increasing while marine fisheries approach the limit of what it can produce. In Sweden, the seafood consumption per capita is higher than the European and world average but the current dietary advice is to increase consumption. Freshwater fisheries have in general been paid less attention in food security discussions. Carp fishes (Cyprinidae) in Sweden have lost their historical value and are currently, both understudied and underutilized. Here we use a combined environmental assessment approach to examine the environmental sustainability of current and potential cyprinid fisheries. We found that current commercial fisheries for Swedish cyprinids in lakes have an average carbon footprint of 0.77 kg CO_2_e per kg of edible product, substantially smaller than most of the popular marine and terrestrial protein sources consumed in Sweden today. This could be even lower if cyprinid resources were better utilized than currently. The cyprinids however exhibited different vulnerability to fishing pressure and are today associated with data deficiencies. Hence, it is currently uncertain how much food for human consumption they can contribute to. Improved consumer interest and management attention is needed, but to the Swedish diet, cyprinids offer a promising opportunity for future more sustainable and nutritious food systems.

## Introduction

The need to shift towards more sustainable food systems advocate for new and innovative approaches (Gordon et al. 2017). Examples include improving resource use efficiency and finding solutions to the current distance between consumers and producers from globalized markets. Aquatic resources could play an important role to future food and nutrition security (Godfray et al. 2010; Tacon and Metian 2013). However, current supply has high demand and is extensively traded, affecting e.g. affordability (FAO 2018). Growth of aquaculture has been vital, considering that marine capture fisheries has not increased since the 1980s; on the other hand, further increase in production may lead to competition of feed resources (Troell et al. 2014). Even so, seafood consumption is often recommended to increase due to important nutritional characteristics—but it is unclear how this could be done sustainably (Thurstan and Roberts 2014).

Freshwater fisheries production is one component of aquatic resources that has traditionally been paid less attention to in terms of contribution to food security (McIntyre et al. 2016; Deines et al. 2017; Lynch et al. 2017) and could potentially add to resource availability. In particular in industrialized countries, that often import the majority of seafood consumed from countries with undernourishment (Smith et al. 2010), freshwater resources are not given adequate consideration as a food resource in terms of management systems needed (Arlinghaus et al. 2002; Dudgeon et al. 2006). There is thus little information available to quantitatively determine sustainable exploitation levels (Lorenzen et al. 2016), and risk-based assessments such as productivity susceptibility analysis have almost exclusively been applied in a marine context (Hordyk and Carruthers 2018). Small-scale fisheries with passive gears, such as those often used in freshwaters, are also understudied in terms of energy use per kilo landing, while the data available exhibit large variability (Parker and Tyedmers 2015). Fuel use during fishing is the key driver behind the carbon footprint of products from capture fisheries (Ziegler et al. 2016; Avadí and Fréon 2013). Therefore, including freshwater products in assessments of healthy and sustainable diets is overall compromised (e.g., Hallström et al. 2019; Tilman and Clark 2014).

Sweden represents an interesting case study related to addressing the role of freshwater fisheries in future food systems. Swedish consumption of seafood is higher than the global and European average (Borthwick et al. 2019), but below the dietary advice of two to three portions per week recommended by the National Food Agency (NFA) in Sweden (NFA Sweden 2019). To reach the recommended level (2.5 portions a week), an increase of 3.75 kg per person and year is required (assuming a portion size of 125 g). However, around 78% of products consumed in Sweden today is imported, most from marine fisheries and aquaculture from around the globe, even if the Swedish government has a food strategy to increase level of self-sufficiency (Borthwick et al. 2019; GoV 2016). Swedish aquaculture production is marginal and has today challenges with strict environmental legislation and high production costs, with only a few commercial actors. However, over 40,000 km^2^ is covered by freshwater (SCB 2019a) while freshwater fisheries represented only 0.7% of Sweden’s reported commercial landings in 2018 (SwAM 2018, 2019a). What role could increased utilization of freshwater fisheries in Sweden play for improved food and nutrition security?

Current freshwater landings in Sweden mainly comprise of a few popular freshwater fishes, such as salmonids (Salmonidae), pikeperch *Sander lucioperca*, perch *Perca fluviatilis* and pike *Esox lucius*, which are fully utilized or even in need of recovery according to the yearly account of status of Swedish fisheries resources (SwAM 2019b). However, there are also populations of virtually unutilized carp species (Cyprinidae). Latest advice recommends an increase in fishing for bream *Abramis brama*, the only only carp species included in the yearly resource assessment, but with caution. Several species of carp were consumed in Sweden up until the middle of the 20th century but have over time been more or less abandoned in the process of industrialization, urbanization and expansion of marine fisheries (Bonow et al. 2013). They are still an important resource in many other countries, including neighboring countries. Today, they are in Sweden mainly being fished for other reasons than for human consumption, such as bait fish and reduction fisheries in lakes to remove nutrients as a remedy against eutrophication (Bonow et al. 2013; Hansson et al. 1998). This implies that there is very limited data available to inform on stock status and sensitivity to fishing pressure (Östman et al. 2016).

To fully identify the net effect of different options for future sustainable diets, or ‘rewiring the food system’ (sensu Gordon et al. 2017), an integration of environmental assessments is needed that is capable of including both potential local ecological risks and offstage effects (Pascual et al. 2017). As an example, while carbon footprints (estimates of greenhouse gas emissions through life cycle assessment (LCA)) are commonly used as proxies for sustainability (e.g. Tilman and Clark 2014), other tools are needed to address local ecological risks of fishing (Ziegler et al. 2016), such as ecological risk assessments (Hobday et al. 2011). The two tools may be seen as complementary to each other in assessing pressures from capture fisheries. This has been demonstrated for the Australian fisheries for Patagonian toothfish *Dissostichus eleginoides*, where local ecological risks were assessed as low whereas the carbon footprint was comparatively high (Hornborg et al. 2018); more case studies may be useful to map potential conflicts between different aspects of sustainability and contribute to discussions on sustainable diets.

The objective of this study is to evaluate Swedish fisheries of cyprinids as a food resource compared to other more popular food items consumed in Sweden today. This is done by assessing the carbon footprint combined with the potential risks posed for the different carp species based on vulnerability to fishing pressure, i.e., a combined approach to environmental assessment. The overall aim is to form a basis for discussing the potential of cyprinids in sustainable food systems.

## Materials and Methods

### Life Cycle Assessment

The carbon footprint was quantified through a LCA approach, a tool commonly used to quantify the environmental pressures and resource use of different products throughout its lifecycle (Ness et al. 2007). The goal was to assess the carbon footprint of the current and potential (defined as 100% retainment of discarded cyprinids) Swedish freshwater cyprinid fishing to enable comparison with (1) other fisheries, based on Parker et al. (2018); (2) other top seafood items consumed in Sweden (Borthwick et al. 2019); and (3) Swedish beef, pork, chicken and brown beans based on RISE Climate Database (2018). The functional unit (FU), i.e., the unit to which all of the inputs and outputs are related to, was defined as 1 kg of edible carp fish meat to be comparable with RISE (2018). The edible yield was collected from FAO (FAO 1989) which states the same yield for all cyprinids (54% of live-weight, based on that of common carp *Cyprinus carpio*).

The system boundaries, i.e., the processes included in the study, covered the production and combustion of fuel for current commercial and recent reduction fisheries in Swedish lakes, up to the point of landing. Transport of the fish to market is excluded. This may add more to emissions than what is generally seen (low contribution of the transports) because the supply chain is today not optimized due to the small-scale nature of the current fisheries. On the other hand, the fishery in at least one of the lakes (Bolmen) has no addition from the supply chain since it is locally prepared and consumed. Only emissions from fuel production and combustion during fishing were considered, while production of boats, fishing gear and packaging was excluded. This was motivated from that the fuel used during the fishing phase generally dominates the carbon footprint of seafood from capture fisheries (Ziegler et al. 2016). Allocation of emissions between species in landings was based on mass; alternative allocation such as economy-based is difficult since the market for cyprinids to a large extent is potential rather than existing. Results will also be less influenced by markets over time. In processing, all emissions are allocated to the edible part of the fish (no utilizations of by-products are known). Cleaning and filleting of the fish were assumed to be done by hand due to the small scale of the freshwater fisheries, and hence no additional resource use and emissions was assigned to this stage.

Emission factors from fuel combustion was collected from the Swedish fuel and biofuel institute (SPBI 2010). It is assumed that all motors fueled by petrol use “Engine petrol without ethanol” (emission factor 2.36 kg CO_2_e/L) whereas by diesel “Diesel – Mk1” (emission factor 2.54 kg CO_2_e/L). Emissions from upstream production of the fuel came from the Ecoinvent database in the LCA software tool SimaPro. This background data accounts for emissions from production of fuel, such as oil field exploration, crude oil production, long distance transportation, oil refining and regional distribution. Emissions were converted from being stated per kg to liters using densities corresponding to fuels at SPBI (2010).

To compare the carbon footprints to global fisheries, the data collected had to be treated according to the method used by Parker et al. (2018). Non-fuel related inputs (such as production of boats and gear) were added as a theoretical estimate based on earlier studies (25% of the total GHG-emissions up to landing of the fish). Fuel use per kg live weight was used, thus making the carbon footprint calculated corresponding to all freshwater fishes landed using the evaluated fishing method.

Data on the current commercial pond net fishery (the fishing method contributing to 88% of cyprinid landings of mainly bream; SwAM 2019a) was collected through semi-structured interviews. A first suggestion of fishers was obtained from the Swedish Lake Fishery Association, adding further fishers through “snow-balling technique” where respondents were asked to suggest others to interview. Two of the Swedish big lakes were included, Vänern and Mälaren, and one smaller lake, Bolmen. In the two larger lakes efforts were made to obtain answers from fishers distributed throughout the lakes’ areas. Two large lakes were excluded, namely Vättern and Hjälmaren. Vättern was originally considered as a potential candidate but was excluded since the nutrient poor lake holds a relatively small cyprinid population, with all the commercial catches already being used in the lakes’ crayfish fishery (J. Fogel, County Administrative Board Stockholm, Pers. Comm.). A total of 14 commercial fishers provided data for one year of fishing (2018). Furthermore, data from five reduction fishing occasions in two lakes was extracted from published reports (Sandström 2011; Annadotter et al. 2013). Additional data on reduction fisheries was obtained from interviews with three reduction fishers from 20 fishing occasions in eight different lakes (between 2010 and 2018). The lakes included were located in the southern part of Sweden, more specifically the regions Skåne (Finjasjön, Häckebergasjön, Oppmannsjön, Ringsjön and Sövdesjön), Småland (Södra Bergundasjön, Trummen and Växjösjön) and Uppland (Vallentunasjön and Östhammarfjärdarna). The samples are seen as representative for the fishing practices since the percentage of cyprinids in landings in the commercial fisheries were approximately the same in the sample and the whole fleet, and data on reduction fisheries covered 40% of all the recent reduction fisheries granted funds from SwAM as Local Water Management Projects (SwAM 2019c). A Kruskal–Wallis one-way analysis of variance (Kruskal and Wallis 1952) was done to determine if there were any significant differences between the carbon footprint of fish from different lakes or fishing methods.

### Productivity Susceptibility Analysis

Productivity-susceptibility analysis (PSA) is a screening analysis to assess the potential and relative vulnerability of a species to a fishery based on a set of attributes related to its productivity (based on life history parameters such as life span) and susceptibility to impacts from fishing activities (based on parameters such as overlap of fishing effort and species distribution), with different approaches used today around the world (Hordyk and Carruthers 2018). The assessment is performed by categorizing a number of attributes of the species and fishery into low/medium/high through a score from 1 to 3. The two average values for the species productivity and susceptibility to the fishery are then combined to form an overall vulnerability *V* by calculating the Euclidean distance from the origin of the two numbers:

1 $${V} = \sqrt {P^2 + S^2}$$

where productivity *P* is the average risk score for all productivity attributes and susceptibility *S* is the average of the multiplicative risk scores for all susceptibility *S* attributes (ranging between 1.41 and 4.24, where *V* < 2.64 equals to low risk and *V* > 3.18 high respectively). The method does not provide insights on the actual status of a species but gives an idea of how potentially vulnerable it is to fishing relative to other species based on its biological properties, useful to determine which ones should be prioritized to be investigated further.

The PSA method used here was based on Hobday et al. (2007, 2011). This method utilizes a precautionary approach to data-deficiency in the scoring of attributes, where an attribute is associated to high-risk if data is lacking or of poor quality. Species included were all native Swedish cyprinid species based on the Encyclopedia of the Swedish Flora and Fauna (Kullander et al. 2012), i.e., excluding five introduced species. This was motivated from that they do not belong to the native fauna and is thus of less conservation concern; in total 16 species were included. Three main target species of the current commercial pound net fisheries were also included in the analysis for comparison: pike *Esox lucius*, perch *Perca fluviatilis* and pikeperch *Sander lucioperca*. The same set of productivity attributes were used as in Hobday et al. (2011; Table 1). Cut-offs for low-medium-high productivity was based on the distribution of all native freshwater species registered in Swedish lakes (37 species found at the Swedish Species Initiative 2019, excluding sub-species), following the recommended approach. The productivity attributes were primarily collected from the Encyclopedia of the Swedish Flora and Fauna (Kullander et al. 2012). When data was lacking (on for example trophic level, maturity size) FishBase (2019) was used. When ranges were provided, the value resulting in the lowest productivity score (i.e., highest risk) was used in order to be precautionary. For details on all life history parameters and risk levels used see Online resource 1. The total productivity score for each cyprinid was calculated as the average risk value for all attributes.

**Table 1:** Life history attributes and productivity cut-offs used based on Swedish freshwater fishes

Productivity attribute	Low productivity (high risk)	Medium productivity (medium risk)	High productivity (low risk)
Age at maturity (years)	>6	4–5	<4
Maximum age (years)	>21	12–20	<10
Fecundity (eggs per year)	<8000	8000–80,000	>100,000
Maximum size (cm)	>60	30–60	<30
Size at maturity (cm)	>30	14–30	<14
Trophic level	>3.7	3.2–3.7	<3.2
Reproductive strategy	Live bearer, sex change	Demersal egg layer	Broadcast spawner

Susceptibility scores were based on the data collected from fishers during the interviews (fishing depths, post capture mortality), together with information found on FishBase (2019) on species depth range and fishing regulations by SwAM (HVMFS 2014:22) on mesh sizes (Table 2). In a few cases, data was extracted from reports published after finished reduction fishery efforts (Sandström 2011; Annadotter et al. 2013). Further information on the fisheries, such as additional methods utilized only in reduction fisheries, was collected from interviews with fishers. Since freshwater fish is restricted by geographic barriers (different lakes) there is a high chance of occurrence of local stocks. The use of a stock structure proxy (as presented by Hobday et al. (2007) was thus deemed most appropriate for the Availability score (set to 3, or high risk). There was no information found on depth ranges for the cyprinids, nor habitat overlap of species and fisheries, thus giving the attribute Encounterability a value of 3 (high risk). Cut-off values for selectivity was following the approach for gillnet, i.e., based on mesh size relative to size for maturity/total size (e.g. high risk equals to sizes >2 × mesh size). The mesh size of the pound nets is generally very small, but passive entrapment gear is by law required to have escape openings with a diameter of 60 mm (HVMFS 2014:22). This diameter was considered the mesh size of the commercial pound nets. Post capture mortality cut-off attribute was set to 3 (high risk) in the current fisheries if the species represented a considerable part (more than 10%) of the total pound net landings (SwAM 2018). Other species were given a score of 2 (medium risk) since they are discarded alive and have a high chance for survival based on fishing method. A multiplicative approach was used for calculating the average susceptibility score following Hobday et al. (2011), except for the reduction fishery where the overall susceptibility score for the cyprinids to was assumed to be 3 (high) since the fishing aims at removing as much cyprinid biomass as possible.

**Table 2:** Susceptibility attributes and cut-offs for low-medium-high-risk based on Hobday et al. (2011) and assumptions made in this study

Attribute	Options	This study		
		High susceptibility	Medium susceptibility	Low susceptibility
Availability	Two alternatives, (1) overlap of species range with fishery (in %) or (2) global distribution	Endemic/sub-population	North hemisphere	Global
Encounterability	Two alternatives, (1) habitat- or (2) depth overlap with fishery (%)	High overlap with fishing gear	Medium overlap with fishing gear	Low overlap with fishing gear
Selectivity	Vary by gear type, based on size (at maturity/maximum)	Species > 2 times mesh size	Species 1–2 times mesh size	Species < mesh size
Post-capture mortality	Vary by gear and species	Retained species, or majority dead when released	Released alive	Evidence of post-capture release and survival

### Evaluation of a Potential Future Carp Fishery

For environmental assessment of a potential increased utilization of cyprinids within the current fishery operations in terms of carbon footprint, ecological risks and available volumes it was assumed that:

1. landing the today discarded cyprinids would not require more fishing trips or more fuel per trip;
2. there are no re-catches of the released cyprinids; and
3. all are of sizes of commercial interest.

The risk score for post capture mortality was adjusted to 3 (high risk) for all cyprinid species in the PSA, and the current fuel use was attributed to higher landing volumes than current (100% retainment of today discarded cyprinids) for the carbon footprint assessment.

To scale up to estimate the potential biomass of cyprinids available to future fisheries from all lakes, cyprinid catches was collected from the Swedish lake monitoring database (NORS 2019) and converted to g of cyprinids per m^2^ of net. The largest nets, Bss (‘översiktsnät typ Stora Sjöarna’), were excluded from the estimate, since these were only used in lake Vänern and are disproportionally ineffective in catching cyprinids compared to smaller nets. The abundance from the geographically closest monitoring fishery site was assumed to be the same as on the stated fishing location. Abundance data from 2015 to 2017 was used for the commercial fisheries, whereas for the reduction fisheries, data from the year before the reduction fishing took place was used if available. When not available, the data from the year closest to the fishing occasion was used (two cases dating back to 1997).

Current commercial use was also collected from FishBase (2019) to map commercial interest, as well as status on the 2015 Swedish IUCN Red List assessment (Swedish Species Initiative 2019) as an additional source on potential vulnerability.

## Results

All cyprinids are categorized as least concern (LC) on the Swedish IUCN Red List except for two which are red-listed (near threatened, NT): asp *Aspius aspius* and vimba bream *Vimba vimba* (Table 3). The global commercial use of the cyprinid species varies considerably, with bream *Abramis brama* and crucian carp *Carassius carassius* being highly commercial, while belica *Leucaspius delineatus*, common dace *Leuciscus leuciscus* and gudgeon *Gobio gobio* being of no commercial interest today (FishBase 2019).

**Table 3:** Native cyprinids in Sweden, IUCN Red List status and commercial utilization globally

Common name	Species	Maximum size	IUCN status	Commercial use
Asp	*Aspius aspius*	120 cm	NT	Yes
Belica	*Leucaspius delineatus*	12 cm	LC	No
Bleak	*Alburnus alburnus*	16 cm	LC	Minor
Bream	*Abramis brama*	80 cm	LC	Highly
Chub	*Squalius cephalus*	45 cm	LC	Minor
Common dace	*Leuciscus leuciscus*	35 cm	LC	No
Crucian carp	*Carassius carassius*	64 cm	LC	Highly
Eurasian minnow	*Phoxinus phoxinus*	12 cm	LC	Minor
Gudeon	*Gobio gobio*	13 cm	LC	No
Ide	*Leuciscus idus*	24 cm	LC	Yes
Roach	*Rutilus rutilus*	50 cm	LC	Yes
Rudd	*Scardinius erythrophtalmus*	35 cm	LC	Minor
Tench	*Tinca tinca*	60 cm	LC	Yes
Vimba bream	*Vimba vimba*	50 cm	NT	Minor
White bream	*Blicca bjoerkna*	45 cm	LC	Minor
Zope	*Ballerus ballerus*	45 cm	LC	Minor

Cyprinid landing ratio relative to other species was found to vary considerably between the different lakes, from 0.8% in Mälaren to 57.5% in Bolmen (Table 4), but also between fishers within the lakes. The annual total landings of all fish and total fuel consumption does however not vary as much between lakes. This results in a relatively homogenous average fuel consumption per kg fish landed, between 0.06 and 0.18 liter of fuel per kg live weight (Table 4). Based on current discard rates, cyprinid landings have the potential to increase substantially, which in turn would lead to improved fuel efficiency per kilo landed.

**Table 4:** Inventory results from the commercial fishery with standard deviation, separated by lake

Lake	% Cyprinids in landings	Total landing per fisher (tonnes)	Cyprinid landing per fisher (tonnes)	Potential cyprinid landing per fisher (tonnes)	Fuel use per fisher (m^3^)	Fuel efficiency (liter/live-weight)
Vänern	19.0	18.2 ± 12.6	3.5 ± 6.0	18.8 ± 14.6	2.2 ± 1.2	0.13 ± 0.07
Mälaren	0.8	20.7 ± 6.7	0.2 ± 0.4	8.0 ± 6.2	3.5 ± 1. 4	0.18 ± 0.06
Bolmen	57.5	40	23	32	2.2	0.06
Average	14.1	21.2 ± 10.2	3.0 ± 6.9	13.5 ± 11.9	3.0 ± 1. 4	0.15 ± 0.07

**Fig. 1:**
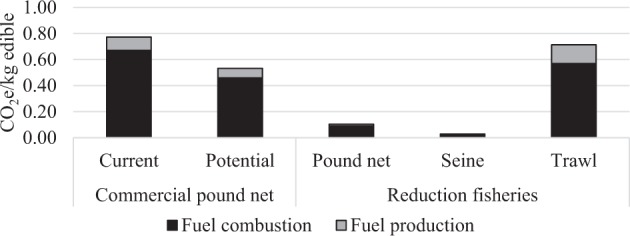
Average carbon footprint of commercial and reduction fisheries (separated by gear) with contribution of fuel combustion and production to the total calculated average carbon footprint

The reduction fisheries all target primarily cyprinids (Table 5). Small perch were occasionally included in the landings from pound nets and seines, making up the missing few percent. The seine and pound net reduction fisheries were all conducted with petrol driven engines, but the trawlers utilized diesel. The carbon footprint of the current commercial fishery in Swedish lakes up to the point of landing was on average 0.77 kg CO_2_e/kg edible, which may decrease by 32% in a future fishery by landing all cyprinids that are discarded today (Fig. 1). The carbon footprint varied between the lakes in the commercial fishery but was not statistically significant (Kruskal–Wallis *H* = 3.51, *p* = 0.17, df = 2). Emissions from reduction fisheries vary significantly depending on gears (Kruskal–Wallis *H* = 18.28, *p* = 0.0001, df = 2), where trawl is similar to commercial pound net fisheries (0.71 kg CO_2_e/kg edible), but seine and pound nets have considerably lower emissions (0.03 kg and 0.10 kg CO_2_e/kg edible respectively).

**Table 5:** Inventory results from the reduction fisheries with standard deviations, divided into groups based on gear type

Gear	% Cyprinids in landings	Total landing per lake (tonnes)	Cyprinid landing per lake (tonnes)	Fuel use per lake (m^3^)	Fuel efficiency (liter/live-weight)
Pound nets	98%	16.3 ± 15.6	15.9 ± 15.4	0.22 ± 0.14	0.02 ± 0.01
Seine	99%	23.7 ± 19.1	23.4 ± 19.2	0.10 ± 0.06	0.005 ± 0.004
Trawl	100%	65.5 ± 22.3	65.5 ± 22.3	7.58 ± 3.01	0.12 ± 0.05
Average	99%	28.8 ± 25.9	28.5 ± 25.9	1.65 ± 3.27	0.04 ± 0.05

**Fig. 2:**
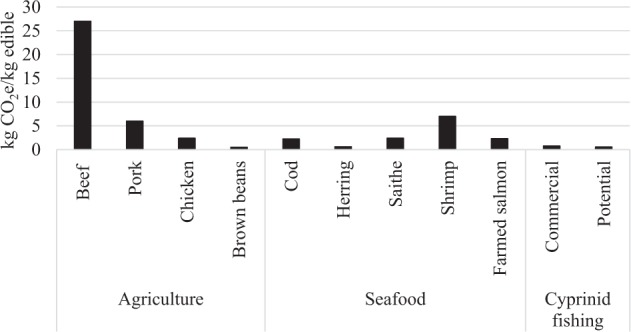
Average carbon footprints for the cyprinids based on this study in relation to other more popular protein sources consumed in Sweden today (based on data from RISE Climate Database 2018)

The carbon footprint of the cyprinids is substantially lower than that of most popular seafoods consumed in Sweden today. Cyprinids caught in the current commercial fisheries contribute to only about a third of the greenhouse gas emissions compared to popular seafood products like Atlantic cod *Gadus morhua*, Saithe *Pollachius virens*, and farmed Atlantic salmon *Salmo salar* (Fig. 2). Herring *Clupea harengus* products are the only ones with a smaller carbon footprint compared to the cyprinids from the current commercial fishery, whereas brown beans has a similar carbon footprint to current commercial cyprinid fisheries.

**Fig. 3:**
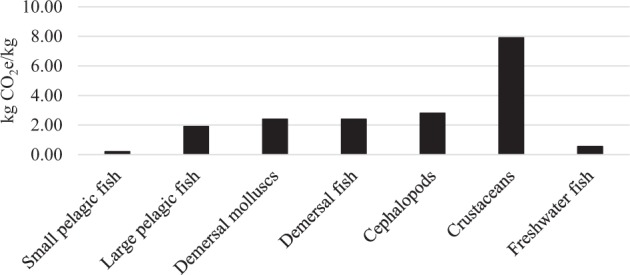
Average carbon footprint for one kg of live-weight fish, including theoretical estimations for upstream production of boats, gear etc., for the Swedish freshwater fisheries examined in this study (labeled as Freshwater fish), relative to marine seafood categories presented by Parker et al. (2018)

In comparison to other fisheries for which there is fuel data, Swedish freshwater pound net and reduction fisheries are amongst the most fuel-efficient fisheries (Fig. 3), with only fisheries of small pelagic fish (<30 cm) being more efficient.

The PSA indicated different risk levels of the species, but none had a higher vulnerability score than current target species (Table 6). Cyprinids had an average productivity score of 1.93 (SD 0.41) whereas the targeted freshwater species had an average productivity score of 2.81 (SD 0.08), i.e. higher risks than any of the individual cyprinid species. The cyprinids had an average vulnerability score of 2.95 (SD 0.48) in the current fisheries, increasing to 3.40 (SD 0.52) in a potential future fishery landing all cyprinids as a result of the increased susceptibility score (post-capture mortality). These estimates are however associated with a lot of uncertainties. Even if most productivity attributes were known (except for size at maturity for two cyprinid species), several susceptibility attributes were given a high-risk based on data deficiency. Out of the 16 considered cyprinid species, four species are considered having low vulnerability, eight medium and four high respectively to the current freshwater pound net fishery. In a future fishery with 100% retainment of cyprinids, the vulnerability scores increase for all species in the family.

**Table 6:** PSA results for the sixteen Swedish cyprinids and three target species in the current commercial fishery and a potential land-all scenario

Group	Species	Productivity	Susceptibility, current	Vulnerability, current	Risk category, current	Susceptibility, potential	Vulnerability, potential	Risk category, potential
Carps	Asp	2.43	2.33	3.36	High	3.00	3.86	High
	White bream	1.86	2.33	2.98	Med	3.00	3.53	High
	Eurasian minnow	1.43	1.43	2.02	Low	1.65	2.18	Low
	Zope	2.00	2.33	3.07	Med	3.00	3.61	High
	Chub	2.14	2.33	3.16	Med	3.00	3.69	High
	Belica	1.57	2.33	2.81	Med	3.00	3.39	High
	Ide	2.71	2.33	3.57	High	3.00	4.05	High
	Bleak	1.29	1.88	2.27	Low	2.33	2.66	Med
	Roach	2.00	2.33	3.07	Med	3.00	3.61	High
	Gudeon	1.29	1.88	2.27	Low	2.33	2.66	Med
	Rudd	1.71	1.88	2.54	Low	2.33	2.89	Med
	Vimba bream	2.14	2.33	3.16	Med	3.00	3.69	High
	Bream	2.29	3.00	3.77	High	3.00	3.77	High
	Crucian carp	2.29	2.33	3.26	High	3.00	3.77	High
	Common dace	1.71	2.33	2.89	Med	3.00	3.46	High
	Tench	2.00	2.33	3.07	Med	3.00	3.61	High
Target	Perch	2.71	3.00	4.05	High	3.00	4.05	High
	Pike	2.86	3.00	4.14	High	3.00	4.14	High
	Pikeperch	2.86	3.00	4.14	High	3.00	4.14	High

## Discussion and Conclusion

The low carbon footprint of current commercial fisheries catching cyprinids in Swedish lakes clearly indicates that it is a promising commodity in this sense compared to most of the commonly consumed protein sources in Sweden today— even compared to most other global fisheries. An even lower carbon footprint per kilo may be achieved simply by utilizing more of the cyprinids that are discarded today. The cyprinids caught in reduction fisheries with seine have an even lower carbon footprint, lower than any other fishery type studied Parker et al. (2018). However, since this fishery is aiming at eradicating fish, the catch efficiency and associated carbon footprint may not be seen as a long-term sustainable food production but rather a underutilized resource; the biomass caught should be further investigated in terms of usefulness as feed or food depending on quality and supply chains enabled.

The carbon footprint does not however, convey information on potential local ecological risks with the fisheries. Jointly considering the carbon footprint results with those of the productivity susceptibility analysis (PSA) is important since the PSA indicated that the different cyprinid species may have different vulnerability to fishing pressure. As a result, even if the carbon footprint of current fisheries (which primarily target bream) would decrease in a fishery where more cyprinids are utilized, potential risks increase, advocating that expansion of fishing should be accompanied by suitable monitoring. PSA is designed to have a precautionary approach and is only an initial screening of relative risks for species to support prioritization of further effort by management and research (Hobday et al. 2011). The assessment was associated with uncertainties and could potentially be improved by further method adaptation to freshwater conditions since life history cues for extinction risk may not be correlated for marine and freshwater fishes (Reynolds et al. 2005; Olden et al. 2007). There is to our knowledge only one example of when PSA has been used to assess the vulnerability in a freshwater context (between populations of Arctic char *Salvelinus alpinus* in Canada; Roux et al. 2011). Even so, none of the species was found to have a potentially higher vulnerability than current target species in Swedish lake fisheries.

The combined carbon footprint and ecological risk-based approach utilized here can illustrate potential trade-offs in sustainability when evaluating new seafood resources from capture fisheries (e.g., freshwater resources, bycatches), and identify different improvement potentials and associated constraints. Combining elements of the two tools, which both are used to support decision making in environmental management, has gained increase interest in recent years (Harder et al. 2015). In a fisheries context, this is the second study utilizing the combined approach, the other being a study on a vastly different fishery in the form of the Australian fisheries for Patagonian toothfish *Dissostichus eleginoides* (Hornborg et al. 2018). The toothfish study showed a development of the fishery from high to low ecological risks through management actions taken, but the opposite for carbon footprint development. The latter is not considered by management authorities but of interest to industry and other societal actors. These results are both opposite and similar to this study. Similar in the sense of being cases of exploitation of new resources, with potential ecological risks higher at start (especially the reduction fisheries) but carbon footprint lower. Opposite, since the assessment indicate low carbon footprint of Swedish cyprinids but higher potential ecological risks due to data deficiency, the latter requiring management effort.

### Cyprinids as a New Resource in the Swedish Food System

Improved utilization of current cyprinid resources offers an opportunity to improve food and nutrition security, given proper measures are taken to minimize potential risks for the species with increased exploitation. From a nutritional perspective, seafood from marine capture fisheries provides important contributions of micronutrients as e.g. shown recently by Hicks et al. (2019). Freshwater fishes were excluded in the analysis due to data deficiencies, but there is no evidence for them having in general lower nutritional value. They may rather have benefits compared to marine species, such as a higher ability to produce longer-chain omega 3 fatty acids out of vegetable oils when farmed (Khalili Tilami, Sampels 2018). Comparing some of the most important nutrients between the freshwater bream (based on the Swedish National Food agency (NFA)) and popular marine fish species (based on Hornborg et al. 2019) show that the cyprinid has higher concentration of vitamin B12, selenium, and niacin than both Atlantic cod *Gadus morhua* and farmed Atlantic salmon *Salmo salar*. The concentration of polyunsaturated fatty acids in bream is lower than in Atlantic salmon, but on the other hand, substantially higher than in Atlantic cod. Therefore, increased utilization of cyprinids in the Swedish seafood diet may improve nutritional security.

In a recent report on nutritional and climate aspects of Swedish seafood consumption (Hornborg et al. 2019), cyprinids were not included as they were deemed irrelevant due to the negligible consumption. In 2017, 52 tonnes of carp fishes were available for consumption in Swedish retail (Borthwick et al. 2019), or 10 grams per person and year (based on the Swedish population in 2019; SCB 2019b). A future fishery when all cyprinids are retained in the commercial fishery and extrapolated to all active fishers in the lakes included in the study could tentatively provide almost 228 tonnes edible product. This would still only represent less than 1% of the increase in fish consumption recommended by the Swedish NFA. If the average current discard level of cyprinids (22%) was extrapolated to all freshwater pound net fishers, including other Swedish lakes, the total landing could be 350 tonnes edible product. This may cause competition between bait and food, especially in lake Vättern, but available volumes could also increase further by including catches from coastal fisheries in the Baltic Sea (were abundance of, e.g., roach is increasing; Ådjers et al. 2006), which were not considered here. Further investigations on potential biomass available and associated risks with exploitation are however required since species composition varies between lakes (Lehtonen et al. 2008), which should also include coastal fisheries.

The potential maximum volume available to human consumption from the reduction fisheries is more difficult to estimate since there is no information on how many lakes that would benefit from reduction fisheries. It would also require more research related to product quality and suitable supply chains to ensure high-quality products. If not suited for human consumption, they should be suitable for other purposes like animal feed, resources much sought after today (Troell et al. 2014). The estimate for potential cyprinid volume available to fishing have in general no information on size- and species-composition, and small individuals may not be of commercial interest. The smallest cyprinids are on the other hand not caught in today’s commercial pound net fisheries, due to the 60 mm escape openings, but are likely caught in reduction fisheries. With these aspects providing uncertainties to the estimates, and by (i) assuming that all Swedish lakes with poor or unsatisfying ecological status would benefit from reduction fishing of cyprinids, and (ii) the catch per hectare in the reduction fisheries included in this study can be extrapolated to all lakes of respective ecological status, a total catch of 3050 tonnes (edible yield) could be possible. This would represent around 8% of the increase in fish consumption recommended by the Swedish NFA. This is however not a stable yearly catch, as reduction fisheries are meant to reduce the cyprinid densities considerably, but some improved utilization than current (at best used for biogas today) should be possible; higher value is also of interest to fishers.

If fishing effort on cyprinids were to increase, monitoring of stock status development is needed where the contribution of this study has only been to identify the potential and relative risks between different species to fishing pressure, i.e., not an assessment on absolute risk. The arguable most important question at this point is however how to re-gain consumer interest in Sweden. This has been lost during the past decades with globalized markets (Bonow et al. 2013). Only three of the sixteen cyprinid species assessed here have no commercial interest in a global perspective, but all have today low consumer interest in Sweden. Some species that should be fully avoided include red-listed species (asp and zope), and belica, the latter on which fishery is also forbidden in Sweden (FIFS 2004:37). Examination of potential occurrence of hazardous substances (such as heavy metals or toxins) in cyprinids caught in different lakes are also needed. This is especially true for the cyprinids caught in reduction fisheries, since lakes with a high inflow of nutrients also often can potentially have a high inflow of other substances as well.

Lastly, seafood is today a highly traded, global commodity (FAO 2018), with pitfalls and promises. The knowledgebase concerning freshwater resources and their vulnerability to effects from climate change differ between countries (Deines et al. 2017; Lynch et al. 2017), calling for increased attention by managers. This may improve by increased consumer interest. A shift back towards improved utilization of local freshwater resources in a country such as Sweden would be an opportunity for improving the current food system (sensu Gordon et al. 2017). This is perhaps especially needed for fish and shellfish since consumers are today distant from the local ecosystem where it is produced and thus fail to see ecosystem effects from demand (Crona et al. 2016). Building resilience in food systems in light of climate change is also urgent, since food production shocks are increasing (Cottrell et al. 2019). Nordic countries have in a European perspective a more vulnerable seafood production sector today than in the past since they are much reliant on temperature-sensitive species—whereas species such as common carp is more tolerant to higher temperatures compared to e.g. salmonids (Blanchet et al. 2019), the top consumed seafood product in Sweden today (Borthwick et al. 2019). Freshwater cyprinids offer an opportunity to Swedish consumers, at this point calling for improved management focus and supply chains.

## Supplementary information

Online Resource1
