# Supplementary material for: Carp (Cyprinidae) Fisheries in Swedish Lakes: A Combined Environmental Assessment Approach to Evaluate Data-limited Freshwater Fish Resources as Food
Source: Environ Manage. 2019 Dec 19;65(2):232–42. doi: 10.1007/s00267-019-01241-z (PMC7007883; doi:10.1007/s00267-019-01241-z)
Supplement: Supplementary file 1 — Online Resource1 [file 267_2019_1241_MOESM1_ESM.docx]

# Online Resource 1

Table 1 Life history parameters used in the study to for productivity attributes and risk scores for susceptibility.

| Common name | Scientific name | Productivity: life history attributes used | | | | | | | Susceptibility: risk scores used | | | | |
| --- | --- | --- | --- | --- | --- | --- | --- | --- | --- | --- | --- | --- | --- |
|  |  | Maturity age | Max age | Fecundity | Max size | Maturity size | Trophic level | Reproductive strategy | Availability | Encounterability | Selectivity | Post-capture mortality, current | Post-capture mortality, potential |
| Asp | *Leuciscus aspius* | 5 | 20 | 80 000 | 120 | 55 | 4.5 | DS | 3 | 3 | 3 | 2 | 3 |
| White bream | *Blicca bjoerkna* | 4 | 20 | 100 000 | 45 | 12 | 3.2 | DS | 3 | 3 | 3 | 2 | 3 |
| Eurasian minnow | *Phoxinus phoxinus* | 1 | 13 | 1 000 | 12 | 5.1 | 3.2 | DS | 3 | 3 | 1 | 2 | 3 |
| Zope | *Ballerus ballerus* | 5 | 20 | 25 000 | 45 | 27 | 3.2 | DS | 3 | 3 | 3 | 2 | 3 |
| Chub | *Squalius cephalus* | 6 | 22 | 200 000 | 45 | 30 | 2.7 | DS | 3 | 3 | 3 | 2 | 3 |
| Belica | *Leucaspius delineatus* | 1 | 8 | 2 000 | 12 |  | 3.2 | DS | 3 | 3 | 3 | 2 | 3 |
| Ide | *Leuciscus idus* | 7 | 23 | 190 000 | 100 | 24 | 3.8 | DS | 3 | 3 | 3 | 2 | 3 |
| Bleak | *Alburnus alburnus* | 3 | 9 | 10 000 | 16 | 10 | 2.7 | DS | 3 | 3 | 2 | 2 | 3 |
| Roach | *Rutilus rutilus* | 5 | 20 | 200 000 | 50 | 14 | 3 | DS | 3 | 3 | 3 | 2 | 3 |
| Gudeon | *Gobio gobio* | 3 | 8 | 8 000 | 13 | 9.3 | 3.1 | DS | 3 | 3 | 2 | 2 | 3 |
| Rudd | *Scardinius erythrophthalmus* | 3 | 20 | 200 000 | 35 | 8.1 | 2.9 | DS | 3 | 3 | 2 | 2 | 3 |
| Vimba bream | *Vimba vimba* | 6 | 20 | 300 000 | 50 | 25 | 3.3 | DS | 3 | 3 | 3 | 2 | 3 |
| Bream | *Abramis brama* | 6 | 30 | 300 000 | 80 | 20 | 3.1 | DS | 3 | 3 | 3 | 3 | 3 |
| Crucian carp | *Carassius carassius* | 4 | 30 | 100 000 | 64 |  | 3.1 | DS | 3 | 3 | 3 | 2 | 3 |
| Common dace | *Leuciscus leuciscus* | 3 | 12 | 30 000 | 35 | 17.9 | 2.9 | DS | 3 | 3 | 3 | 2 | 3 |
| Tench | *Tinca tinca* | 3 | 20 | 300 000 | 60 | 20 | 3.7 | DS | 3 | 3 | 3 | 2 | 3 |
| Perch | *Perca fluviatilis* | 7 | 22 | 20 000 | 61 | 16.8 | 4.4 | DS | 3 | 3 | 3 | 3 | 3 |
| Pike | *Esox lucius* | 5 | 30 | 200 000 | 150 | 37.6 | 4.1 | DS | 3 | 3 | 3 | 3 | 3 |
| Perchpike | *Sander lucioperca* | 5 | 23 | 1 000 000 | 100 | 39 | 4 | DS | 3 | 3 | 3 | 3 | 3 |
